# Modelling autosomal dominant optic atrophy associated with *OPA1* variants in iPSC-derived retinal ganglion cells

**DOI:** 10.1093/hmg/ddac128

**Published:** 2022-06-02

**Authors:** Paul E Sladen, Katarina Jovanovic, Rosellina Guarascio, Daniele Ottaviani, Grace Salsbury, Tatiana Novoselova, J Paul Chapple, Patrick Yu-Wai-Man, Michael E Cheetham

**Affiliations:** UCL Institute of Ophthalmology, 11-43 Bath Street, London EC1V 9EL, UK; UCL Institute of Ophthalmology, 11-43 Bath Street, London EC1V 9EL, UK; UCL Institute of Ophthalmology, 11-43 Bath Street, London EC1V 9EL, UK; UCL Institute of Ophthalmology, 11-43 Bath Street, London EC1V 9EL, UK; Department of Biology, University of Padua, and Veneto Institute of Molecular Medicine, Padua 35129, Italy; Centre for Endocrinology, William Harvey Research Institute, Barts and the London School of Medicine and Dentistry, Queen Mary University of London, Charterhouse Square, London EC1M 6BQ, UK; Centre for Endocrinology, William Harvey Research Institute, Barts and the London School of Medicine and Dentistry, Queen Mary University of London, Charterhouse Square, London EC1M 6BQ, UK; Centre for Endocrinology, William Harvey Research Institute, Barts and the London School of Medicine and Dentistry, Queen Mary University of London, Charterhouse Square, London EC1M 6BQ, UK; UCL Institute of Ophthalmology, 11-43 Bath Street, London EC1V 9EL, UK; Moorfields Eye Hospital NHS Foundation Trust, London EC1V 2PD, UK; Cambridge Eye Unit, Addenbrooke’s Hospital, Cambridge University Hospital, Cambridge CB2 0QQ, UK; Cambridge Centre for Brain Repair and MRC Mitochondrial Biology Unit, Department of Clinical Neurosciences, University of Cambridge, Cambridge CB2 0PY, UK; UCL Institute of Ophthalmology, 11-43 Bath Street, London EC1V 9EL, UK

## Abstract

Autosomal dominant optic atrophy (DOA) is the most common inherited optic neuropathy, characterized by the preferential loss of retinal ganglion cells (RGCs), resulting in optic nerve degeneration and progressive bilateral central vision loss. More than 60% of genetically confirmed patients with DOA carry variants in the nuclear *OPA1* gene, which encodes for a ubiquitously expressed, mitochondrial GTPase protein. OPA1 has diverse functions within the mitochondrial network, facilitating inner membrane fusion and cristae modelling, regulating mitochondrial DNA maintenance and coordinating mitochondrial bioenergetics. There are currently no licensed disease-modifying therapies for DOA and the disease mechanisms driving RGC degeneration are poorly understood. Here, we describe the generation of isogenic, heterozygous *OPA1* null induced pluripotent stem cell (iPSC) (OPA1+/−) through clustered regularly interspaced short palindromic repeats (CRISPR)/Cas9 gene editing of a control cell line, in conjunction with the generation of DOA patient-derived iPSC carrying *OPA1* variants, namely, the c.2708_2711delTTAG variant (DOA iPSC), and previously reported missense variant iPSC line (c.1334G>A, DOA plus [DOA]+ iPSC) and CRISPR/Cas9 corrected controls. A two-dimensional (2D) differentiation protocol was used to study the effect of *OPA1* variants on iPSC-RGC differentiation and mitochondrial function. OPA1+/−, DOA and DOA+ iPSC showed no differentiation deficit compared to control iPSC lines, exhibiting comparable expression of all relevant markers at each stage of differentiation. OPA1+/− and *OPA1* variant iPSC-RGCs exhibited impaired mitochondrial homeostasis, with reduced bioenergetic output and compromised mitochondrial DNA maintenance. These data highlight mitochondrial deficits associated with *OPA1* dysfunction in human iPSC-RGCs, and establish a platform to study disease mechanisms that contribute to RGC loss in DOA, as well as potential therapeutic interventions.

## Introduction

The inherited optic neuropathies (IONs) comprise a range of genetically diverse disorders characterized by the preferential loss of retinal ganglion cells (RGCs), optic nerve degeneration and progressive bilateral visual loss. Autosomal dominant optic atrophy (DOA) is the most common ION, with an estimated minimum prevalence of 1 in 25 000 (1). DOA typically presents with insidious visual loss during the first two decades of life, bilateral central scotomas and optic disc pallor caused by the loss of RGCs within the papillomacular bundle (2). Most patients with DOA experience progressive visual loss and approximately half will be registered legally blind by the fifth decade of life (2). Although visual loss and optic atrophy are the defining features of DOA, a subgroup of patients develop a multisystemic syndromic form of the disease, known as DOA plus (DOA+), with additional extraocular features including sensorineural hearing loss and ataxia (3,4).

More than 60% of all genetically confirmed cases of DOA are caused by variants in the *optic atrophy 1* gene (*OPA1*; 3q28-q29; OMIM 605290; 2), which encodes a ubiquitously expressed dynamin-related mitochondrial GTPase that is anchored to the inner mitochondrial membrane (IMM) (5). More than 500 unique *OPA1* variants have been identified, with more than 80% believed to be pathogenic (6). The majority of variants are thought to generate null alleles, causing premature termination codons (PTC) or loss of function variants that result in haploinsufficiency (7); a hypothesis further supported by the identification of patients carrying large-scale *OPA1* deletions (8,9). Missense variants within the *OPA1* GTPase domain are associated with a higher risk of developing DOA+ due to a putative dominant-negative effect (4). Despite the advances in our understanding of the genetic basis of DOA, the specific sensitivity of RGCs to *OPA1* mutations, especially given the high levels of gene expression within other neuronal tissues, such as the brain and auditory neurons (10), remains unclear.

OPA1 is a multifunctional protein that plays a key role in mitochondrial network organization, promoting IMM fusion and regulating cristae morphology (11–13). As expected, OPA1 dysfunction is associated with increased mitochondrial network fragmentation and aberrant IMM structure (11,13–16). OPA1 also functions as a key regulator of mitochondrial bioenergetic output (17,18) with DOA-associated variants shown to impair mitochondrial oxidative phosphorylation (OXPHOS) and adenosine triphosphate (ATP) synthesis (14,15,19). Reduced mitochondrial bioenergetics is likely driven by mitochondrial respiratory chain complex instability, primarily of complex I and V (ATP synthase), reduced efficiency of mitochondrial electron flux and disturbed IMM cristae structure (19,20).

Furthermore, OPA1 regulates mitochondrial homeostasis via the maintenance of mitochondrial DNA (mtDNA) integrity, through the interaction of *OPA1* with mtDNA displacement loops. Decreased expression of *OPA1* significantly affects mtDNA copy number and distribution throughout the mitochondrial network (20,21). Analysis of mtDNA quality in DOA patient-derived samples and mouse models of DOA demonstrated reduced mtDNA copy number and the accumulation of somatic mtDNA mutations when compared to wild-type (WT) cells (19,22–26). As such, *OPA1* disease-associated variants exert a detrimental effect on mitochondrial homeostasis, since mtDNA encodes for key components required for mtDNA replication and mitochondrial respiratory chain assembly. In addition, OPA1 dysfunction has been shown to disrupt mitophagy (27) and increase cellular susceptibility to apoptotic stimuli (19,28).

A key challenge for DOA research is the inherent difficulty in obtaining patient retinal tissue samples to effectively evaluate the pathogenic disease mechanisms driving RGC loss (1). *In vitro* disease modelling advances have increased our ability to generate physiologically relevant DOA models to further understand *OPA1* disease variants. This is of particular significance given that RGCs and the optic nerve are the solely affected tissue in most patients. Therefore, studies of non-physiologically relevant cell lines may not allow the complete pathological picture to be evaluated. Similarly, rodent models of DOA also have inherent drawbacks since rodent retinas do not contain a macula with a defined papillomacular bundle, which is particularly vulnerable in DOA.

Recently, we demonstrated that clustered regularly interspaced short palindromic repeats (CRISPR)/Cas9 gene correction of DOA-associated induced pluripotent stem cells (iPSCs) can restore mitochondrial homeostasis, improving mtDNA maintenance, ATP production and reducing susceptibility to apoptosis (29). A number of other studies have also demonstrated the feasibility of generating DOA patient-derived iPSC carrying *OPA1* variants (30–32); however, they did not investigate the disease mechanisms associated with OPA1 dysfunction. To date, there has been one report of iPSC-derived-RGC DOA modelling, showcasing an *OPA1* c.2496 + 1G>T splice site mutation predicted to cause mis-splicing of *OPA1* transcripts (33). Analysis of *OPA1* mutant iPSCs demonstrated increased cellular apoptosis when compared to WT controls, as well as an RGC differentiation deficiency, with an inability to form neural precursor cells (NPCs) and ultimately RGCs. However, *OPA1* variant iPSCs could be driven towards an NPC and RGC lineage with the addition of noggin or oestrogen during the differentiation process. Therefore, further study of *OPA1* variants in *in vitro* RGCs is required to clarify the mechanisms that contribute to their preferential loss in DOA.

Here, we describe the generation of an isogenic *OPA1* heterozygous knockout (KO) iPSC line through CRISPR/Cas9 gene editing alongside the generation of DOA patient-derived iPSC. We validated the generation of *in vitro* iPSC-derived RGCs (iPSC-RGCs) harbouring *OPA1* variants, demonstrating that *OPA1* variants have no detrimental impact on the ability of iPSC to differentiate towards an RGC lineage. In iPSC-derived RGCs, *OPA1* variants impaired iPSC-RGC mitochondrial function, including respiratory output and mtDNA maintenance.

## Results

### Generation of *OPA1* knockout and patient-derived iPSC

**Figure 1:**
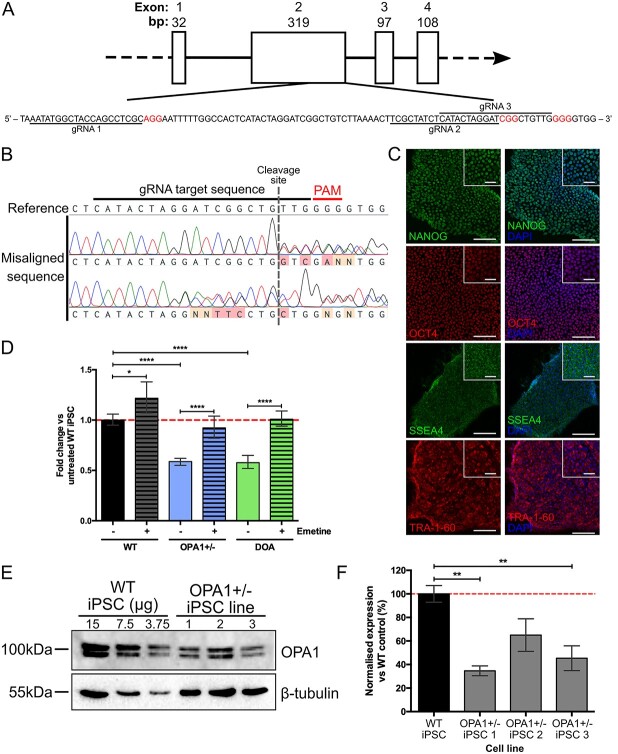
Generation of isogenic *OPA1* heterozygous knockout iPSC. (**A**) Schematic of *OPA1* exons 1–4 and expanded exon 2 sequence showing location of gRNA target sequences (underlined) and protospacer adjacent motif sites (red). (**B**) Sequence alignment of isolated iPSCs clones to the WT *OPA1* sequence enables detection of CRISPR/Cas9 induced mutations (red/orange mis-aligned bases) surrounding the CRISPR/Cas9 gRNA cut site. (**C**) Isogenic OPA1+/− iPSCs demonstrate nuclear localization of ESC markers NANOG and OCT4 with membrane/cytoplasmic localization of SSEA4 and TRA-1-60. Scale bars = 100 and 40 μm for inset. (**D**) Expression of total *OPA1* in CRISPR/Cas9 edited OPA1+/− and patient-derived DOA iPSCs, with and without emetine, a NMD inhibitor. *OPA1* expression was normalized to the geometric mean of *GAPDH* and *ACTIN* before normalization to WT expression. *n* = 4 individual iPSC RNA preparations, ^*^^*^^*^^*^P < 0.0001. (**E**) Analysis of OPA1+/− iPSCs OPA1 protein expression via western blot. OPA1 antibody detects short and long isoforms of OPA1 at ~ 100 kDa. β-tubulin reference protein, 55 kDa, used for normalization of OPA1 expression. (**F**) Three individual OPA1+/− iPSC cell lines show significantly reduced levels of OPA1 protein expression compared to the WT control iPSC, after normalization to reference protein β-tubulin. Bars represent mean ± SEM, *n* = 3 biological replicates from 3 independent western blots, ^*^P < 0.05, ^*^^*^P < 0.01, ^*^^*^^*^P < 0.001.

In order to study the effect of OPA1 reduction on RGC health, we established an isogenic heterozygous OPA1 KO cell line through the simultaneous reprogramming and CRISPR/Cas9 gene editing of an otherwise healthy fibroblast cell line to model haploinsufficiency *OPA1* variants. CRISPR/Cas9 guide RNAs (gRNAs) were designed to target *OPA1* exon 2 (Fig. 1A) and simultaneously nucleofected into WT cells along with episomal reprogramming vectors. Induced pluripotent stem cell colonies were manually isolated and *OPA1* mutation was confirmed through alignment of the *OPA1* exon 2 to the WT sequence (Fig. 1B), with 7/52 iPSC clones demonstrating heterozygous *OPA1* gene editing (*OPA1*+/− iPSC). No homozygous OPA1 KO clones were generated. In addition to the isogenic cell lines, we generated iPSC from a patient-derived fibroblast cell line carrying a heterozygous c.2708_2711delTTAG variant in *OPA1* exon 27 (DOA iPSC), which is the most common *OPA1* variant in DOA and is confirmed to cause OPA1 haploinsufficiency (7,34). Sequencing of the region surrounding *OPA1* exon 27 confirmed the presence of the c.2708_2711delTTAG in both patient-derived fibroblasts and iPSCs (Supplementary Material, Fig. S1A). Following the generation of both iPSC lines immunofluorescence (IF) staining confirmed the expression of pluripotency markers, NANOG, OCT4, SSEA4 and TRA-1-60 in OPA1+/− iPSCs (Fig. 1E) and DOA iPSCs (Supplementary Material, Fig. S1B), indicating successful induction of endogenous stem cell circuitry. The pluripotent status of both OPA1+/− and DOA iPSCs was further analysed using the TaqMan scorecard assay, which compares gene expression of naïve iPSCs and following the undirected differentiation of embryoid bodies. This confirmed the pluripotent nature of both OPA1+/− and DOA iPSC lines (Supplementary Material, Fig. S1C, D).

Following the establishment of OPA1+/− iPSC line, *OPA1* expression was quantified through quantitative polymerase chain reaction (qPCR) and western blot analysis. Quantitative PCR analysis demonstrated an approximate 45% reduction in total *OPA1* transcripts in the OPA1+/− iPSC line when compared to isogenic WT cells, comparable to that of DOA iPSCs that also showed an approximate 45% reduction in expression (Fig. 1D). Inhibition of nonsense-mediated decay (NMD) by emetine, a translation inhibitor that blocks NMD, significantly increased *OPA1* expression in both OPA1+/− and DOA iPSCs when compared to untreated iPSCs (Fig. 1D). Western blot analysis of 3 independent OPA1+/− iPSC lines, all harbouring PTC mutations in *OPA1* exon 2, confirmed an average reduction in OPA1 protein expression of 48% ±8.8% (Fig. 1E, F). Following WB analysis, OPA1+/− iPSC line 3 was selected for further experimentation. Finally, the predicted top 10 off-targets analysed through Sanger sequencing. No off-target effects were determined in the selected OPA1+/− cell line (Supplementary Material, Fig. S2).

### *In vitro* differentiations of iPSC-RGCs

**Figure 2:**
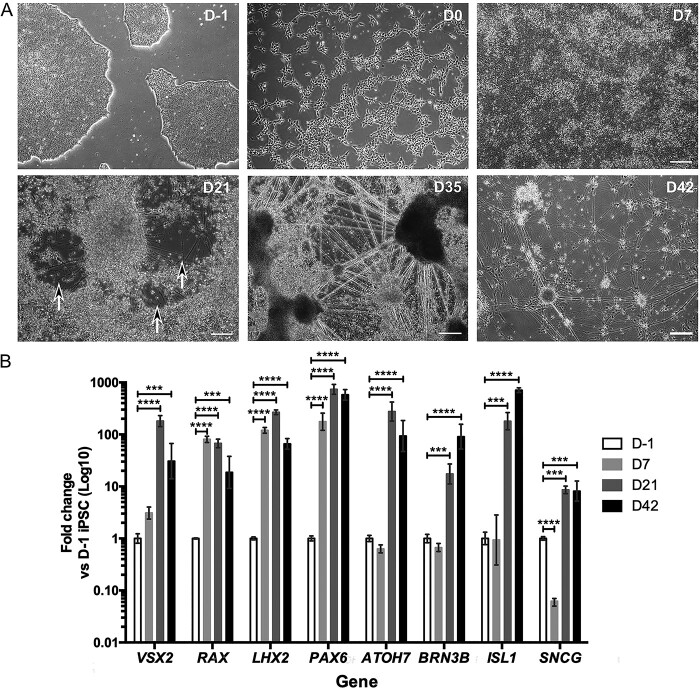
*In vitro* differentiation of iPSC-RGCs. (**A**) Representative phase contrast images of iPSC-RGC differentiation showing cellular morphology at distinct time points during retinal differentiation. D21, black arrows highlight regions with axonal projections. Scale bars = 250 μm. (**B**) Temporal gene expression during iPSC-RGC differentiation at day (D) D-1, D7, D21 and D42 of control RGC differentiation cultures to determine expression of key RGC genes involved in early development (*VSX2*, *RAX*, *LHX2*, *PAX6*), the specification of RGCs (*ATOH7*) and terminal RGC differentiation (*ISL1*, *BRN3B*, *SNCG*). Gene expression was first normalized to the geometric mean of *GAPDH* and *ACTIN*, and then normalized to D-1 undifferentiated iPSC. Bars represent mean expression ± SEM. *n* = 3–4 RNA samples per time point from 3–4 independent differentiations. ^*^^*^^*^P < 0.001, ^*^^*^^*^^*^P < 0.0001.

Following the generation of OPA1+/− and DOA iPSCs we optimized the *in vitro* differentiation of iPSC-RGCs using a previously established small-molecule 2D differentiation protocol (35), which enabled the rapid generation of iPSC-RGCs through a stepwise differentiation method. In order to determine the efficiency of iPSC differentiation towards a retinal and RGC lineage, a time course of retinal and RGC associated gene induction was determined in comparison to undifferentiated WT iPSCs (Fig. 2A). WT iPSCs were initially dissociated to single cells and plated in E8 Flex media, followed by transition to N2B27 media on day 0 (D0) (Fig. 2A) and addition of small molecule compounds on D1. By D7, iPSCs had expanded, generating a confluent sheet of cells (Fig. 2A) that was associated with a significant induction of important eye field transcription factors (EFTFs) *RAX*, *PAX6* and *LHX2* (Fig. 2B) required for retinal progenitor cell (RPC) development. The EFTF *VSX2* showed a slower onset, with a more moderate increase at D7 (Fig. 2B). EFTF expression was maintained at D21, with a moderate reduction in *RAX* expression and further increases in *VSX2, LHX2* and *PAX6* expression (Fig. 2B).

By D21, axonal projections began to appear in differentiation cultures, which could be identified in cell-free areas as the cells contracted into clusters (Fig. 2A). Additionally, when compared to naïve iPSCs (harvested at D-1), there was significant upregulation of *ATOH7*, a pro-neural gene required for RGC specification that results in RGC depletion when ablated (Fig. 2B) (36,37). By D35, substantial axonal projections had developed (Fig. 2A), coupled with further bunching or contraction of cells into dense clusters. The propensity for presumptive iPSC-RGCs to cluster was used to isolate them from other cells in the culture. D35 cultures were treated with Accutase to dissociate the heterogeneous cell sheet, allowing the removal of non-clustered cells that dissociated into singlets whilst allowing extraction of presumptive iPSC-RGCs, which were resistant to dissociation medium, for replating. Isolated neuronal clusters were subsequently cultured for a further 7 days until D42. At this time point, iPSC-RGC clusters had developed extensive axonal networks (Fig. 2A). Analysis of markers typically associated with post-mitotic, mature RGCs demonstrated a slower induction compared to EFTFs (Fig. 2B), indicative of downstream activation during differentiation. *BRN3B* showed a moderate reduction in expression at D7 when compared to D-1 iPSCs, but was significantly increased at D21, followed by a further increase at D42 (Fig. 2B). In addition, *ISL1*, a *BRN3B* synergistic gene required for RGC development (38,39), showed a statistically significant increase at D21 and a further increase at D42 (Fig. 2B). γ-synuclein (*SNCG*)*,* a gene identified as a specific marker of RGCs (40), was initially reduced at D7, before a significant increase in expression at D21 and D42 (Fig. 2B). Together, these results suggest successful induction of developmental and RGC-specific transcriptional pathways during the course of differentiation.

### *OPA1* mutation does not inhibit RGC specification

**Figure 3:**
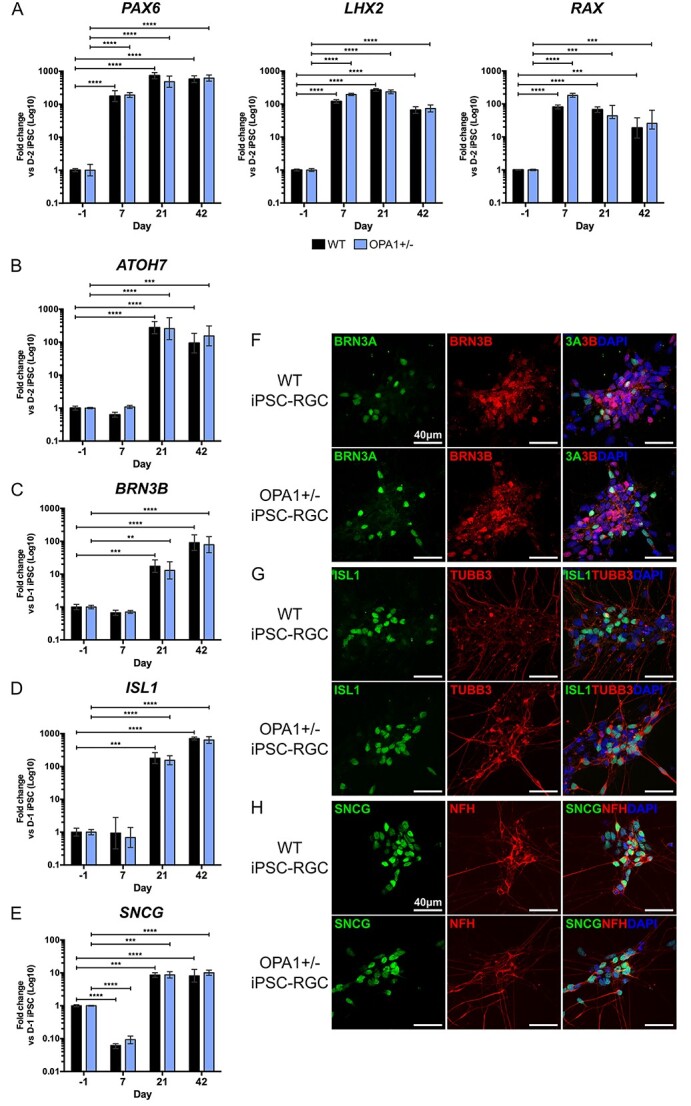
*OPA1* haploinsufficiency does not impair *in vitro* iPSC-RGC differentiation. (**A–D**) Temporal qPCR analysis of key RGC transcription factors *PAX6*, *RAX*, *LHX2* and *ATOH7* (A) and terminal RGC genes *BRN3B* (B), *ISL1* (C) and *SNCG* (D) at D-1, D7, D21 and D42 in WT control and OPA1+/− differentiations. Gene expression was first normalized to the geometric mean of *GAPDH* and *ACTIN* expression, and then normalized to D-1 undifferentiated iPSC to determine fold change. Bars represent mean expression ± SEM. *n* = 3–4 RNA samples per time point from 3–4 independent differentiations. ^*^^*^P < 0.005, ^*^^*^^*^P < 0.01, ^*^^*^^*^^*^P < 0.001. (**E–G**) Immunofluorescent analysis of OPA1+/− and control iPSC-RGCs at D42 to confirm expression of terminal differentiation markers. RGCs express transcription factors BRN3A and BRN3B (E), ISL1 (F) and SNCG (G) and cytoskeletal proteins neurofilament and βIII tubulin (TUBB3) (F,G). Cell nuclei identified with DAPI. Scale bars = 40 μm.

**Figure 4:**
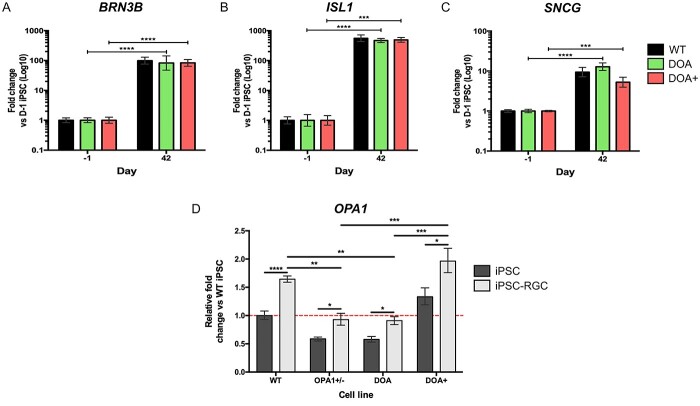
Characterization of *in vitro* iPSC-RGCs generated from DOA patient-derived iPSC. (**A–C**) Temporal qPCR analysis of key RGC transcription factors *BRN3B* (A), ISL1 (B) and *SNCG* (C) at D-1, D7, D21 and D42 in WT control, DOA and DOA+ iPSC differentiations. DOA and DOA+ iPSC demonstrated no significant impairment in terminal RGC gene expression when compare to WT iPSC. Gene expression was first normalized to the geometric mean of *GAPDH* and *ACTIN* expression, and then normalized to D-1 undifferentiated iPSC to determine fold change. Bars represent mean expression ± SEM. *n* = 3–4 RNA samples per time point from 3–4 independent differentiations. ^*^^*^^*^P < 0.001, ^*^^*^^*^^*^P < 0.0001. (**D**) Quantification of total *OPA1* expression in OPA1+/−, DOA and DOA+ iPSC-RGCs via qPCR. All cell lines demonstrate significant upregulation of *OPA1* in iPSC-RGC cultures when compared to naïve D-1 iPSC. OPA1+/− and DOA iPSC-RGCs demonstrate significantly reduced *OPA1* expression compared to WT iPSC-RGCs. *OPA1* expression was first normalized to the geometric mean of *GAPDH* and *ACTIN* expression, and then normalized to D-1 undifferentiated WT iPSC to determine fold change. Bars represent mean expression ± SEM. *n* = 3–4 RNA samples per time point from 3–4 independent differentiations. ^*^P < 0.05, ^*^^*^P < 0.01, ^*^^*^^*^P < 0.001 ^*^^*^^*^^*^P < 0.0001.

Given the previous reports suggesting *OPA1* variants affect the ability for stem cell differentiation (28,40), we investigated whether *OPA1* haploinsufficiency impairs the ability of iPSC to RGC differentiation. As such, OPA1+/− iPSCs were differentiated in parallel with isogenic WT iPSCs to allow for the specific comparison of cell lines that differ solely at the *OPA1* genetic locus. Analysis of genes required for RPC development and early RGC specification demonstrated no significant differences in expression between OPA1+/− and WT iPSCs at any differentiation time point (Fig. 3A, B). Similarly, there were no significant differences in expression of the RGC markers *BRN3B*, *ISL1* and *SNCG* (Fig. 3C–E), and by D42 there was significant expression of all three markers with WT differentiations demonstrating 99.1 ± 27.1, 559.4 ± 144 and 9.4 ± 2.6-fold change for *BRN3B*, *ISL1* and *SNCG*, respectively, when compared to naïve iPSCs (Fig. 3C–E). Similarly, OPA1+/− differentiations demonstrated 74.6 ± 25.8, 593.4 ± 143.1 and 9.2 ± 4.8-fold change, respectively, *BRN3B*, *ISL1* and *SNCG* when compared to OPA1+/− iPSCs (Fig. 3C–E). IF staining also confirmed similar distribution of BRN3A, ISL1 and SNCG positive cells between OPA1+/− and WT iPSC-RGCs (Fig. 3F–H), also demonstrating expression of BRN3B (Fig. 3F), neuronal cytoskeletal proteins β-III tubulin (TUBB3; Fig. 3G) and neurofilament heavy chain (Fig. 3H). To confirm that *OPA1* variants do not have a detrimental impact on iPSC-RGC differentiation, we used the patient-derived DOA iPSC line and a previously reported iPSC line harbouring the c.1334G>A missense variant, which was established from a patient with a DOA+ phenotype (DOA+ iPSCs) (29). Quantitative PCR analysis supported the data from the isogenic cell lines, demonstrating no significant differentiation shortfalls for either DOA or DOA+ iPSCs when compared to WT controls, with comparable expression of *BRN3B, ISl1* or *SNCG* in their respective iPSC-RGC cultures (Fig. 4A–C).

Following quantification of iPSC-RGC differentiation, *OPA1* expression was analysed in iPSC-RGCs through qPCR, demonstrating a significant increase in *OPA1* levels during differentiation. Isogenic WT and OPA1+/− iPSC-RGCs exhibited a 60% and 40% increase in *OPA1* expression when compared to naïve iPSCs (Fig. 4D). OPA1+/− iPSC-RGCs maintained a similar expression profile to their iPSC counterparts, demonstrating an approximate 50% reduction in *OPA1* mRNA levels when compared to WT iPSC-RGCs (Fig. 4D). As with the isogenic iPSC pair, both patient-derived DOA and DOA+ iPSC-RGCs demonstrated significant induction of *OPA1* expression, showing increases of approximately 55% and 47%, respectively, when compared to naïve iPSC (Fig. 4D). Again, DOA iPSC-RGCs maintained the *OPA1* expression pattern demonstrated in naïve iPSC, with an approximate 45% reduction in total *OPA1* mRNA when compared to WT iPSC-RGCs (Fig. 4D). DOA+ iPSC-RGCs demonstrated a significant increase of *OPA1* when compared to both OPA1+/− and DOA iPSC-RGCs (Fig. 4D); however, this was not significantly higher than WT iPSC-RGCs. Collectively, these results suggest that *OPA1* variants alter the expression of *OPA1* in iPSC-RGCs, but these reductions do not impact on the ability of iPSC to differentiate *in vitro* into iPSC*-*RGCs. Importantly, these results also highlight the higher levels of expression of *OPA1* within RGC populations, potentially indicating why RGCs are selectively vulnerable to *OPA1* expression changes caused by *OPA1* variants.

Finally, in order to confirm the maturation of *in vitro* iPSC-RGCs and test for the presence of other cell types, reverse transcriptase polymerase chain reaction (RT-PCR) analysis was used to determine the expression of genes required for neuronal function and genes specific for retina cell lineages (Supplementary Material, Fig. S3A). RT-PCR for mature neuronal markers demonstrated significant upregulation of cytoskeletal components (*TAU*, *NFL*, *NFM*, *TUBB3*) neuronal RNA splicing factors (*NEUN*, *ELAVL3*), axonal guidance (*NRP1*, *ROBO2*, *DCC*) and genes associated with synaptic function (*SYNTAXIN*, *SNAP25*, *VAMP2*; Supplementary Material, Fig. S3A). In addition, we found no evidence of other post-mitotic retinal cell lineages when D42 iPSC-RGC cultures were assessed by RT-PCR for non-RGC retinal associated genes, most notably photoreceptors (*RCVRN*, *ARR3*, *NRL*) and Müller glial cells (*CRALBP*), within the terminal differentiation culture (Supplementary Material, Fig. S3B).

### OPA1 dysfunction inhibits RGC bioenergetics and mtDNA maintenance

**Figure 5:**
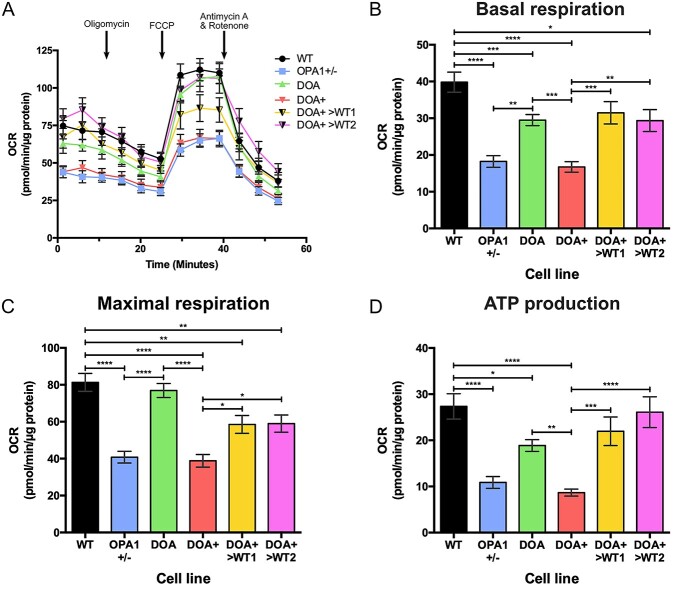
Seahorse bioenergetic analysis of 2D-RGCs. (**A**) Oxygen consumption rate (OCR) was determined using the Seahorse XFe96 Analyzer for WT, OPA1+/−, DOA, DOA+, DOA+>WT1 and DOA+>WT2 iPSC-RGCs. 1.5 μM oligomycin, 1 μM FCCP and 0.5 μM antimycin A and 0.5 μM rotenone were injected at the indicated time points. Symbols represent mean OCR ± SEM. (**B–D**) Analysis of Seahorse bioenergetic profiles reveals the respiratory phenotype of *OPA1* variant iPSC-RGC cultures. *OPA1* variant cell lines have lower levels of basal respiration when compared to WT cells (B). Isogenic *OPA1* mutant cell lines show reduced levels of maximal respiration, the level of OCR after FCCP injection, when compared to WT cell lines (C). ATP production is reduced in *OPA1* variant cell lines, and restored with CRISPR/Cas9 gene correction of the DOA+ variant (D). Bars represent mean OCR ± SEM. *n* = 6 experimental replicates. Bars represent mean OCR ± SEM. ^*^P < 0.05, ^*^^*^P < 0.01, ^*^^*^^*^P < 0.001, ^*^^*^^*^^*^P < 0.0001.

Previous reports investigating *OPA1* variants in non-RGC cell lines have demonstrated a significant impact on mitochondrial function and bioenergetics (20,23,41). Therefore, we investigated the effect of OPA1 dysfunction in iPSC-RGCs through Seahorse bioenergetic analysis, utilizing the OPA1+/−, DOA iPSC and the previously generated DOA+>WT iPSC lines (29). Generation and analysis of iPSC-RGC bioenergetic profiles (Fig. 5A) demonstrated that *OPA1* variants caused significant deficits in basal respiration in iPSC-RGCs, with all three *OPA1* variant lines showing statistically significant reductions in basal oxygen consumption rate (OCR) levels when compared to WT controls (Fig. 5B). DOA iPSC-RGCs demonstrated a significantly higher level of basal respiration than both OPA1+/− and DOA+ iPSC-RGCs (Fig. 5B). CRISPR/Cas9 correction of the DOA+ variant also had a significant impact on basal RGC respiration, with both DOA+>WT1 and >WT2 iPSC-RGCs demonstrating a significantly higher basal OCR when compared to DOA+ iPSC-RGCs (Fig. 5B). However, DOA+>WT2 iPSC-RGCs had reduced basal respiratory rate when compared to control WT iPSC-RGCs (Fig. 5B).

In addition, analysis of maximal respiration demonstrated significant deficits for both OPA1+/− and DOA+ iPSC-RGCs (Fig. 5C). DOA iPSC-RGCs showed no significant reduction in maximal respiration when compared to WT iPSC-RGCs and also demonstrated significantly higher levels than both OPA1+/− and DOA+ iPSC-RGCs (Fig. 5C). Similarly, CRISPR-corrected DOA+>WT1 and >WT2 iPSC-RGCs displayed significantly higher levels of maximal respiration compared to DOA+ iPSC-RGCs, although this was significantly lower than WT iPSC-RGCs (Fig. 5C). Analysis of ATP production further confirmed the effects of the *OPA1* variants on RGC bioenergetics, with OPA1+/−, DOA and DOA+ iPSC-RGCs having significantly reduced levels of ATP production when compared to WT iPSC-RGCs (Fig. 5D), whilst DOA iPSC-RGCs also demonstrated higher levels of ATP production than DOA+ RGCs (Fig. 5D). Both CRISPR-corrected DOA+>WT1 and >WT2 iPSC-RGCs both demonstrated significantly higher levels of ATP production when compared to DOA+ iPSC-RGCs and this was not significantly different to the WT control, unlike both basal and maximal respiration (Fig. 5D).

**Figure 6:**
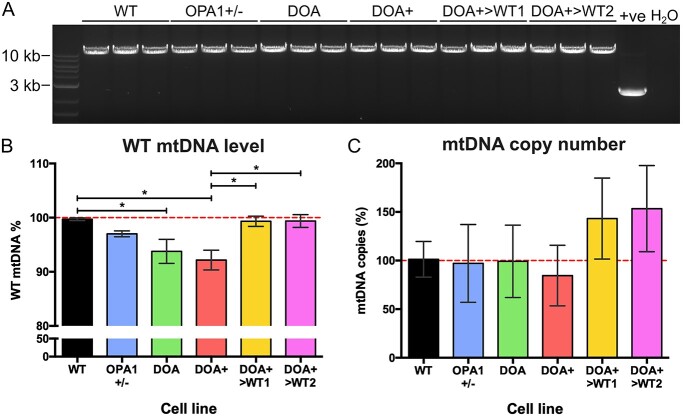
Analysis of mtDNA quality in iPSC-RGCs. (**A**) A 9.9 kb region spanning the major arc of mtDNA was expanded via LR-PCR. No major mtDNA deletions were detected in *OPA1* variant iPSC-RGCs. Each lane represents an individual DNA sample from 3 separate iPSC-RGC differentiations. +ve denotes cybrid cell line with known mtDNA deletion. H_2_O = water only control. (**B**) Quantitative PCR analysis of iPSC-RGCs revealed reduced levels of WT mtDNA in DOA and DOA+ iPSC-RGCs, whilst CRISPR/Cas9 corrected DOA+>WT1 and >WT2 returned to WT levels. Bars represent mean ± SEM. *n* = 5–8 individual iPSC-RGC DNA samples. ^*^P < 0.05, ^*^^*^P < 0.01. (**C**) mtDNA copy number analysis of 2D-RGCs demonstrates no significant alterations in *OPA1* variant cell lines. Bars represent mean ± SEM.

In addition to quantifying the effect of *OPA1* mutation on iPSC-RGC bioenergetic output, we explored the effect of *OPA1* mutation on mtDNA maintenance in iPSC-RGCs. Previous studies have established that dysfunctional OPA1 results in mtDNA instability (23–26). As such, we investigated the effect of *OPA1* variants on mtDNA maintenance in iPSC-RGC cultures via long-range PCR (LR-PCR) and qPCR, enabling detection of both large- and small-scale deletions alongside mtDNA copy number changes. LR-PCR did not detect any large-scale mtDNA rearrangements in all iPSC-RGC genotypes, with a clear band produced at 10 kb and no further PCR products observed in the gel when compared to a heteroplasmic mitochondrial cybrid cell line that had significant mtDNA deletion (Fig. 6A). Following LR-PCR, qPCR analysis demonstrated significantly reduced levels of WT mtDNA in both DOA and DOA+ iPSC-RGCs, indicative of minor deletions or mutations within the qPCR region, when compared to WT iPSC-RGCs (Fig. 6B), with 92.7 ± 19.3% and 91.8 ± 22.3% versus 100.0 ± 15.5% WT mtDNA levels, respectively (Fig. 6B). No significant differences were detected in OPA1+/− iPSC-RGCs. However, CRISPR/Cas9 gene-corrected DOA+>WT1 and >WT2 iPSC-RGCs showed significant restoration of WT mtDNA levels when compared to DOA+ iPSC-RGCs, with DOA+>WT1 and DOA+>WT2 iPSC-RGCs showing 99.3 ± 19.7% and 101.4 ± 43.2% WT mtDNA levels, respectively (Fig. 6C). No significant changes of mtDNA copy number were detected in iPSC-RGCs (Fig. 6C).

## Discussion

Current cell models of IONs lack the tissue specificity required to understand the disease mechanisms associated with RGCs, the primary affected cell type. The advent of iPSC technology, CRISPR/Cas9 gene editing and advances in *in vitro* differentiation have significantly increased our ability to accurately and specifically model IONs, which have previously proven challenging. Here, we report the generation of isogenic heterozygous *OPA1* KO iPSCs derived from an otherwise healthy fibroblast cell line using simultaneous CRISPR/Cas9 gene editing and cellular reprogramming. In addition, we produced DOA patient-derived iPSCs harbouring the most common pathogenic *OPA1* variant (c.2708_2711delTTAG) seen in patients with DOA. Subsequently, we investigated the ability for *OPA1* variant cell lines to generate *in vitro* iPSC-RGCs using a previously described *in vitro* differentiation protocol, which demonstrated that these *OPA1* iPSC lines were fully able to differentiate towards an RGC fate. Using this model, we demonstrate that iPSC-RGCs harbouring *OPA1* variants have significantly impaired mitochondrial bioenergetic output and reduced mtDNA maintenance, highlighting important disease mechanisms associated with DOA that have previously not been characterized in human RGC populations.

Previous studies have suggested that *OPA1* variants impede neuronal development in *in vitro* differentiation models (28,40). Here, isogenic *OPA1*+/− and WT cell lines were differentiated simultaneously through a modified 2D differentiation protocol. Importantly, isogenic cell lines remove inter-individual genetic heterogeneity from differentiation systems, allowing the specific study of isolated genetic changes and their impacts on developmental processes. A previous study of DOA patient-derived iPSCs carrying an *OPA1* splice variant were unable to generate RGCs due to an inability to generate NPCs (33), a process that is, in part, regulated by *PAX6* and *LHX2* (42,43). In contrast, temporal qPCR analysis in our study demonstrated significant upregulation of genes associated with NPC development during the early stages of differentiation, with significant upregulation of the EFTFs *PAX6*, *LHX2* and *RAX*. In addition, analysis of isogenic embryonic stem cells (ESCs) carrying a heterozygous CRISPR/Cas9 *OPA1* variant also confirmed the ability for OPA1+/− cells to successfully generate NPCs (28).

It should be noted that the previous study suggested the addition of Noggin, a BMP antagonist, as a factor capable of rescuing the differentiation of RGCs for their DOA patient-derived iPSC lines (33). Conversely, the protocol used here, which is based on a previously published protocol (35), alongside studies by others (44,45), includes dorsomorphin, another BMP antagonist, or an equivalent within their differentiation protocols for the generation of RGCs. Studies of animal models have also confirmed the requirement of BMP inhibition during retinal development. Treatment of ectodermal explants or stem cells extracted from frog (*Xenopus*) and mouse with noggin induces retinal fate and drastically increased EFTF expression, including *Pax6* and *Lhx2* (46,47). Thus, it can be argued that noggin-induced BMP inhibition is likely to be an essential requirement for RGC differentiation.

Furthermore, analysis of terminal differentiation markers, *ATOH7*, *BRN3B*, *ISL1* and *SNCG*, which are essential for RGC development (48), demonstrated no significant differences for OPA1+/− iPSCs, which was further corroborated by analysis of two independent DOA patient-derived iPSC lines. This demonstrates that regardless of the type of variant, OPA1 dysfunction does not impact differentiation ability. The lack of a clear developmental deficit was also suggested by the TaqMan Scorecard assay, demonstrating the competence of OPA1+/− iPSCs for differentiating into the three primary germ layers when compared to WT iPSC and a reference panel of iPSC and ESCs.

In addition, studies of iPSC-derived neurons have highlighted potential disease mechanisms of *OPA1* variants associated with neuronal populations. Analysis of iPSC-derived dopaminergic neurons and ESC-derived neurons, harbouring *OPA1* variants or CRISPR/Cas9 *OPA1* edits, suggested OPA1 dysfunction reduces neuronal OXPHOS, increases mitochondrial fragmentation and impairs stem cell neuronal differentiation (28,33,49,50). Although these studies have provided some insight into the specific disease mechanisms associated with neuronal loss, further study of *OPA1* mutations in RGCs is required to clarify the mechanisms that contribute to their preferential loss in DOA.

A number of studies have generated iPSCs carrying *OPA1* variants (30–32); however, to date no study has analysed the bioenergetic consequences of *OPA1* variants in human iPSC-RGCs. Previous analysis of patient-derived iPSCs carrying an *OPA1* variant, associated with Parkinson's disease, showed that when differentiated into dopaminergic neurons or into NPCs they demonstrated significant reductions in basal respiration, maximal respiration and ATP output for haploinsufficient variant cells when compared to WT controls (49,51). Furthermore, knockdown of *OPA1* within *ex vivo* rat primary cortical neurons revealed a significant reduction in basal and maximal respiration, associated with an approximate 70% reduction in protein level (41). The results presented in this study further expand on and concur with these studies by analysing the effect of multiple *OPA1* variant types in human iPSC-RGCs, demonstrating deficiencies in basal respiration and ATP production for all variants, and reduced maximal respiration for OPA1+/− and DOA+ iPSC-RGCs. Importantly, corrected DOA+>WT1 and DOA+>WT2 iPSC-RGCs also demonstrated significant improvements in mitochondrial respiration when compared to DOA+ iPSC-RGCs. It is unclear why the corrected DOA+>WT iPSC-RGCs lines showed lower maximal respiration than the control line. This is unlikely to be related to the gene correction as the iPSCs showed higher maximal respiration than a panel of control iPSCs (29). Therefore, it could reflect some type of compensation in the patient line which is manifesting differently in the iPSC-RGCs or inter-individual variation between iPSC-RGCs.

Crucially, this work demonstrates a previously uncharacterized bioenergetic deficit within human RGCs and thus provides greater understanding of the specific disease mechanisms potentially leading to RGC loss. Due to their unique anatomical morphology, RGCs have high metabolic demands and they depend on efficient OXPHOS for most of their energy requirements (52,53), thus making neuronal populations exquisitely susceptible to OXPHOS deficits and reduced ATP production. Deficits in mitochondrial OXPHOS are commonly associated with both genetic and sporadic neurodegenerative diseases, including Leigh syndrome, Parkinson's disease, and multiple sclerosis (54–56). In particular, RGCs are believed to have remarkably high metabolic needs due to their long axonal pathways and their lack of myelination in the retina before the lamina cribosa (57). It is, therefore, not surprising that both inherited and acquired optic neuropathies, including Leber hereditary optic neuropathy and glaucoma, demonstrate deficits in mitochondrial bioenergetics that drive RGC loss (58–61).

Conversely, a recent study of isogenic OPA1+/− ESCs suggested that heterozygous *OPA1* KO does not impair mitochondrial respiration of ESCs or *in vitro* neurons, which was postulated to be a result of ESCs and *in vitro* neurons retaining approximately 50% OPA1 expression (28). However, both haploinsufficient cell lines within this study showed similar levels of *OPA1* mRNA reduction whilst OPA1+/− iPSCs demonstrated an approximate 50% reduction in protein. Analysis of the mitochondrial networks in the previously reported OPA1+/− ESC demonstrated no clear gross morphological abnormalities, with only moderate changes to cristae structure. However, our previous study of DOA+ iPSCs found significant levels of mitochondrial fragmentation when compared to WT and CRISPR/Cas9 corrected cell lines, which was also in agreement with the study of iPSC-derived neurons (49). This may therefore suggest that due to the lack of mitochondrial morphological changes, the OPA1+/− ESC were unlikely to exhibit bioenergetic alterations. Importantly, the differences between our results and previous studies demonstrate the importance of conducting context-specific modelling for understanding the disease mechanisms associated with DOA disease progression. The data presented here further support dysfunctional mitochondrial bioenergetics as a significant contributor to RGC degeneration. A better understanding of why specific neuronal populations are impacted by *OPA1* variants and the specific downstream effects of energy deficit on neuronal survival could have broader significance for other neurodegenerative diseases associated with RGC loss.

In addition to RGC bioenergetics, the effect *OPA1* variants on mtDNA maintenance was evaluated in iPSC-derived RGC populations. OPA1 is thought to play a role in maintaining the mitochondrial genome by anchoring mtDNA through direct interaction of exon 4b and the mtDNA D-loop (21,62). In accordance with a number of previous studies (23–26), analysis of mtDNA in *in vitro* cultured cells demonstrated a significant reduction in mtDNA WT levels for both DOA and DOA+ iPSC-RGCs. Interestingly, although CRISPR/Cas9 heterozygous KO of OPA1 resulted in a trend to reduce WT mtDNA levels, this was not significant. The lack of significant mtDNA defects in our OPA1+/− cell line suggests that mtDNA defects may be acquired over time and the relatively short period of time our cell line has harboured an *OPA1* defect may not be sufficient for the clonal expansion of mtDNA mutations to high levels (63). However, our previously generated DOA+>WT cell lines demonstrated significantly restored mtDNA quality in iPSC-RGCs. Although a number of previous studies have demonstrated altered mtDNA copy number associated with *OPA1* variants (15,19,22,23,25), in this study we found no significant differences in mtDNA copy number amongst *in vitro* iPSC-RGCs, although the DOA+ line was trending to be reduced. The loss of mtDNA integrity is a common phenotype associated with neurodegenerative diseases, including Parkinson's disease and Alzheimer's disease (64–66). The development of methods to counter mtDNA genome instability may prove a useful strategy for reducing the degeneration of RGCs in patients with DOA (4,25).

Interestingly, one previous study suggested that only *OPA1* mutations associated with DOA+ drive mtDNA defects (27). In our study, both DOA (c.2708_2711delTTAG) and DOA+ (c.1334G>A) variants were associated with decreased WT mtDNA levels, suggesting that both missense and haploinsufficient mutations impaired mtDNA quality control. In addition, restoration of GTPase function through CRISPR/Cas9 gene correction restored WT mtDNA levels, further indicating the importance of a functional GTPase domain in mitochondrial homeostasis (20). Thus, the data presented here support the theory that *OPA1* missense variants within the GTPase domain have a detrimental impact on mtDNA integrity, further supporting the hypothesis that GTPase point mutations exert an enhanced deleterious effect due to a dominant-negative effect.

In conclusion, we have produced a series of OPA1 iPSC lines that model haploinsufficient and potential dominant-negative disease with paired isogenic controls. We have used these cell lines to further define the pathogenic mechanisms associated with DOA. Both OPA1+/− iPSC and DOA patient-derived iPSCs showed no impaired ability to differentiate into RGCs *in vitro.* Importantly, we demonstrate that iPSC-RGCs carrying *OPA1* variants exhibit typical phenotypic hallmarks associated with *OPA1* dysfunction, most notably, impaired mitochondrial bioenergetic output and mtDNA instability. These novel observations obtained from *in vitro* human RGC models of DOA provide further insight into the complex disease mechanisms that precipitate RGC loss and optic nerve degeneration, ultimately causing progressive visual loss. The generation of isogenic cell lines and patient-derived iPSCs provides an invaluable resource to further investigate the pathological consequences of specific *OPA1* variants and importantly, to investigate the potential benefit of new therapies, including gene therapy, for patients with DOA.

## Materials and Methods

### CRISPR/Cas9 gene editing of *OPA1*

Isogenic OPA1 KO stem cells were produced following a simultaneous reprogramming and CRISPR/Cas9 gene-editing protocol (67), by targeting *OPA1* in otherwise healthy human dermal fibroblasts, of neonatal origin (HDFn). Guide RNAs were designed to target *OPA1* exon 2 (Supplementary Material, Table S1) and cloned into the pSpCas9(BB)-2A-GFP (PX458; gift from Feng Zhang; Addgene plasmid #48138), according to a previously described protocol (68). Control HDFn were grown to 90% confluency in fibroblast growth media Dulbecco’s Modified Eagle Medium (DMEM with 10% foetal bovine serum, 1% non-essential amino acids and 1% penicillin–streptomycin; all Gibco) and dissociated with 0.05% Trypsin–EDTA (Gibco). 1 × 10^6^ cells were isolated, resuspended in Nucleofector solution from Cell Line Nucleofector Kit R (Lonza) containing 1 μg of each episomal reprogramming vector (69) and 2 μg of the OPA1 targeting PX458 plasmid. Following nucleofection, cultures were maintained until the presence of iPSC colonies emerged. Individual clones were mechanically isolated and placed into individual wells of a geltrex-coated 12-well plate.

Clonal iPSC lines were subsequently expanded, before DNA extraction using the Wizard SV genomic DNA extraction kit (Promega) following the manufacturer’s instructions. The CRISPR/Cas9 target region was expanded using primers *OPA1* exon 2 (Supplementary Material, Table S2) and analysed by Sanger sequencing to detect the presence or absence of CRISPR/Cas9 induced mutations by aligning the sample sequence data to the *OPA1* reference sequence (ENST00000392438, ensemble.org) on Benchling (Benchling.com). Guide RNA off-target sites (Supplementary Material, Table S3) were predicted using Off-Spotter (https://cm.jefferson.edu/Off-Spotter/) (70), and the top 10 off-targets expanded by PCR (Supplementary Material, Table S2) before Sanger sequencing using the forward primer and alignment on Benchling (Benchling.com) to confirm the presence or absence of mutations.

### Generation of patient-derived iPSC

DOA patient-derived iPSCs were generated as previously described (29), using a skin biopsy taken from an affected individual with isolated optic atrophy and carrying a heterozygous *OPA1* c.2708_2711delTTAG:p.R905^*^ variant. Informed consent was obtained following the tenets of the Declaration of Helsinki. Ethical approval was granted by the Yorkshire and The Humber—Leeds Bradford Research Ethics Committee (REC reference: 13/YH/0310). The presence of the *OPA1* mutation was confirmed in DOA patient-derived iPSCs via Sanger sequencing using primers OPA1-ex27 forward and OPA1-ex27 reverse (Supplementary Material, Table S2).

### iPSC culture

Once generated, iPSCs were maintained in E8 Flex medium (Gibco) on Geltrex coated plates. Induced pluripotent stem cells were passaged using enzyme-free cell dissociation buffer (Gibco) and manual separation twice-weekly, and maintained in a 5% CO_2_ incubator at 37°C.

### *In vitro* differentiation of retinal ganglion cells

Differentiation of iPSC-RGCs was based on a previously published protocol by Sluch *et al*. (35) with minor modifications. Undifferentiated iPSCs were maintained in Geltrex-coated T25 flasks in E8 flex media until the day of differentiation (Day −1). On D-1, iPSCs were washed with phosphate-buffered saline (PBS) and dissociated to single cells using TrypLE Express for 15 min at 37°C, cells were scraped and resuspended in E8 flex media containing 5 mM blebbistatin before seeding onto 1% Matrigel (Corning) coated plates at 52 000 per cm^2^. The day after plating, designated as D0, media was changed for N2B27 media (1:1 mix of DMEM/F12 and Neurobasal media with 1X GlutaMAX Supplement, 1X volume by volume (v/v) antibiotic–antimycotic, 1% N2 supplement and 2% B27 supplement; all Gibco). N2B27 was kept at 4°C for a maximum of 1 week. The following day, D1, small molecules were added to cells in fresh N2B27 media and fed every other day with a complete exchange of N2B27 media, except if a small molecule was required to be added or removed. Small molecules were added on the following days at the final concentrations indicated: Dorsomorphin (1 μM, Stratech) and IDE2 (2.5 μM, Peprotech), for D1 to D6, Nicotinamide (10 mM, Sigma) for D1 to D10, Forskolin (25 μM, Peprotech) for D1 to D30 and DAPT (10 μM, Abcam). Differentiation cultures were maintained 37°C in 5% CO_2_. Upon reaching D35 presumptive RGCs were purified by extracting ‘neuronal clusters’ from the heterogeneous cell culture. Cells were washed with PBS and dissociated with Accutase (Sigma) for 30–45 min at 37°C. Cultures were subsequently resuspended in N2B27 media, triturated and strained twice using a 70 μm nylon strainer (Falcon) to remove single cells. Cell clusters were retained and washed with PBS before resuspension in N2B27 media and centrifugation at 0.1*g.* Afterwards, neuronal clusters were plated on 1% Matrigel in N2B27 media and cultured for a further 5–10 days for analysis.

### RNA extraction and cDNA synthesis

Total RNA was extracted using the RNeasy Mini Kit (QIAGEN) according to the manufacturer’s instructions. First-strand cDNA synthesis was completed using the Tetro cDNA synthesis kit (Bioline) and diluted to a final volume of 50 ng starting RNA per 100 μl H_2_O.

### RT-PCR and qPCR

RT-PCR analysis of cDNA was completed using 2X GoTaq green master mix (Promega) and 0.5 μM forward and reverse primers (Supplementary Material, Table S2) and 5 μl cDNA per reaction. RT-PCR reactions were incubated in a thermocycler for a total of 28–30 cycles, before gel electrophoresis in a 2% agarose gel with Safeview nucleic acid stain (NBS Biologicals).

Quantitative PCR was completed utilizing the SYBR Green method run on a Quantstudio 6 Flex real-time PCR system (Thermofisher). A master mix was prepared containing 10 μl of 2X LabTAQ hi-rox green master mix (Labtech) and 0.4 μM forward and reverse primers (Supplementary Material, Table S2), and combined with 5 μl of cDNA sample, with each sample was loaded in triplicate. Quantitative PCR data were collected in QuantStudio Real-Time PCR software (Applied Biosystems) and raw data were exported to Microsoft Excel. Data were quantified using the delta–delta cycle threshold (Ct) (ΔΔCt) method, using the geometric mean of two reference genes, *GAPDH* and *ACTIN*.

### Immunofluorescence and imaging

Cellular proteins were detected using indirect IF analysis. Samples were washed with 1X (PBS; Oxoid) and fixed for 10 min at room temperature (RT) in 4% paraformaldehyde (ThermoFisher) diluted in PBS. Samples were permeabilized for 15 min at RT in 0.1% triton X-100 (Sigma) in PBS, followed by blocking in block buffer (PBS containing 10% (v/v) normal donkey serum and 0.3% weight by volume bovine serum albumin). Primary antibodies (Supplementary Material, Table S4) were incubated overnight at 4°C in 1:1 block buffer:PBS, negative controls were performed without primary antibodies. After primary incubation samples were washed with PBS and incubated in corresponding AlexaFluor 488 or 555 secondary antibodies in 1:1 block buffer:PBS for 1–2 h at RT. Nuclei were visualized with 4′,6-diamidino-2-phenylindole dilactate (Sigma) for all images. After staining, cells were washed and mounted with Dako fluorescent mounting medium (Dako) or Vectorshield® Vibrance Antifade mounting medium (Vector Laboratories) and left to set overnight at RT. All images were obtained using Carl Zeiss LSM700 or LSM710 laser-scanning confocal microscope (Zeiss) or a Leica TCS SP8 MP confocal microscope (Leica Microsystems). Images were exported from Zen imaging software and prepared using FIJI (imagej.net/Fiji) and InkScape (Inkscape.org).

### Assessment of mtDNA deletion and copy number

To detect possible mtDNA deletions, LR-PCR was used to target the major arc of the mitochondrial genome, as previously described (29).

Quantification of mtDNA deletion levels and mtDNA copy number was conducted as previous (29), using primers designed to enable quantification of mtDNA genes, *MTND1* and *MTDN4,* and nuclear DNA encoded genes, *B2M* and *GAPDH* (Supplementary Material, Table S2). Induced pluripotent stem cell-RGC clusters were seeded onto 1% Matrigel (Corning) coated 24 well plates 5–7 days before DNA extraction. Total DNA was extracted using the Wizard® SV Genomic DNA Purification System following the manufacturer’s instructions. A total of 50 ng DNA was loaded per qPCR reaction, loaded in triplicate. mtDNA copy number analysis was completed using the ΔΔCt method and presented as percentage of control mtDNA copies.

### Mitochondrial bioenergetic assessment

Live assessment of cellular bioenergetics was performed using the Seahorse XFe96 extracellular flux analyser (Seahorse Bioscience) following the manufacturer’s instructions. Induced pluripotent stem cell-RGC clusters were seeded onto 1% Matrigel-coated plates 3–5 days prior to analysis. On the day of experimentation, cell media was changed to XF base media approximately 60 min before analysis and incubated at 37°C in a CO_2_-free incubator. The Seahorse XFe96 machine was calibrated before the measurement of mitochondrial respiration (oxygen consumption rate, OCR). After baseline measurements, cells were treated with 1.5 μM Oligomycin, 1 μM carbonyl cyanide p-triflouromethoxyphenylhydrazone and 0.5 μM rotenone and antimycin-A. Once completed, cells were lysed with radioimmunoprecipitation assay buffer plus 2% proteinase inhibitor cocktail (Sigma) and total protein determined with a Pierce bicinchoninic acid Assay Kit (Thermofisher). Data were subsequently analysed using Wave software (Seahorse Bioscience) and raw data exported to Microsoft Excel.

### Statistical analysis

Statistical analysis was completed using GraphPad Prism 6 software (GraphPad Inc.) using a Student’s *t*-test or one-way analysis of variance with a Tukey’s multiple comparison test. Results are represented as arithmetic mean ± standard error of the mean (SEM) unless otherwise stated.

## Supplementary Material

HMG-2022-CE-00215-R1_Sladen_et_al_Supplementary_data_ddac128
