# Supplementary material for: Modelling autosomal dominant optic atrophy associated with OPA1 variants in iPSC-derived retinal ganglion cells
Source: Hum Mol Genet. 2022 Jun 2;31(20):3478–93. doi: 10.1093/hmg/ddac128 (PMC9558835; doi:10.1093/hmg/ddac128)
Supplement: HMG-2022-CE-00215-R1_Sladen_et_al_Supplementary_data_ddac128 [file hmg-2022-ce-00215-r1_sladen_et_al_supplementary_data_ddac128.zip › HMG-2022-CE-00215-R1_Sladen_et_al_Supplementary_data_ddac128.pdf]

Modelling autosomal dominant optic atrophy associated with *OPA1* variants in iPSC-derived retinal ganglion cells Sladen et al

Supplementary figures and tables

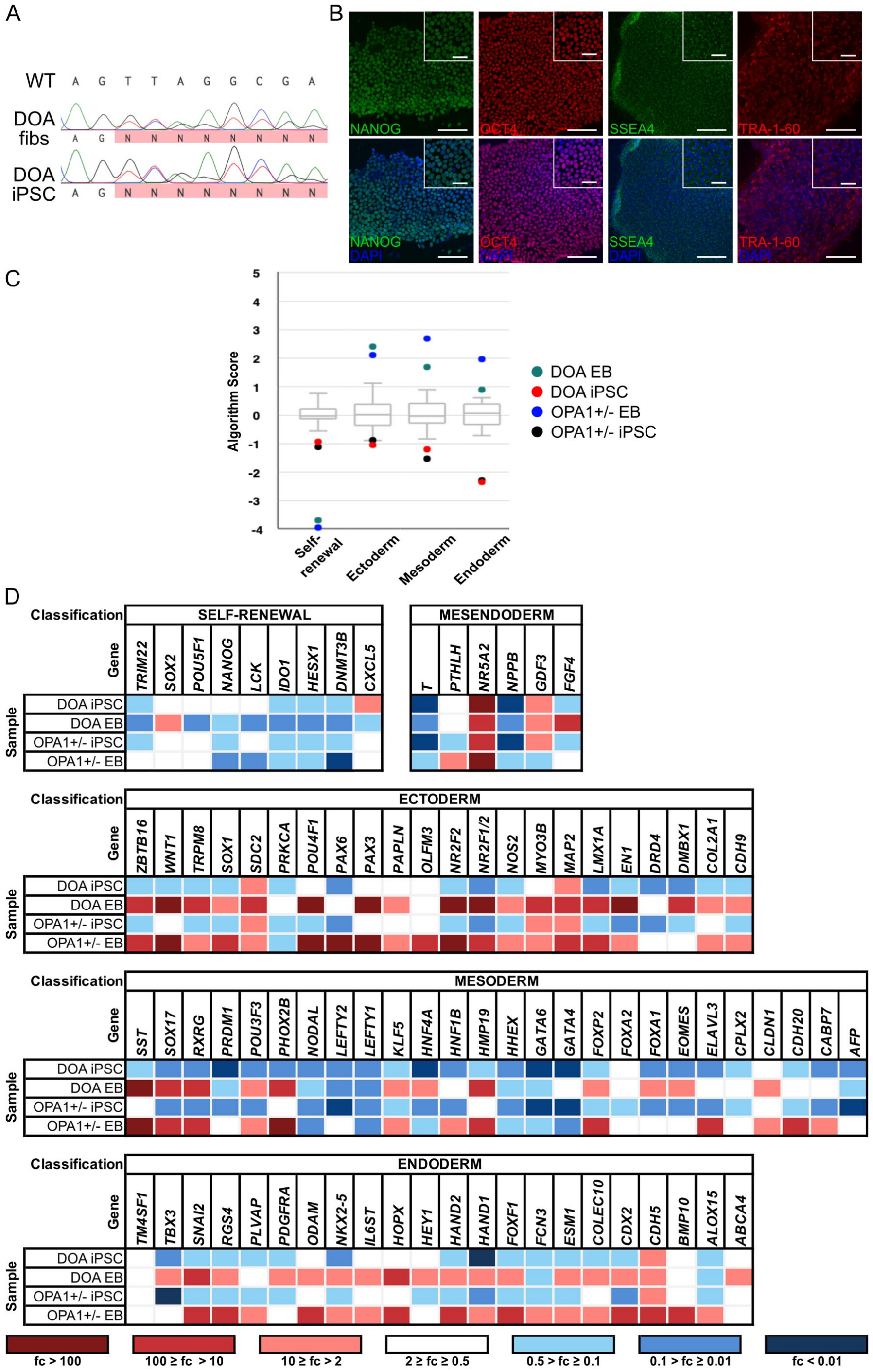

Figure S1. Generation of DOA patient derived iPSC carrying an *OPA1* c.2708_2711delTTAG variant.

(A) Patient-derived fibroblasts carrying a c.2708_2711delTTAG variant in *OPA1* were reprogrammed to iPSCs by nucleofection of non-integrating episomal plasmids. Sanger sequencing of generated iPSCs confirmed the presence of the 4 base pair deletion variant within both patient-derived fibroblasts and iPSCs.

(B) Immunofluorescent staining of DOA iPSCs confirmed the expression of embryonic stem cell associated proteins NANOG, OCT4, SSEA4 and TRA-160. Scale bars 100µm and 40µm for inset.

(C) TaqMan hPSC Scorecard assay algorithm analysis confirms the pluripotency of OPA1+/- and DOA iPSCs. Expression of germ layer markers is significantly upregulated following undirected embryoid body (EB) differentiation, when compared to naïve iPSCs. Box and whisker plot demonstrating average expression of self-renewal and germ layer genes in the Scorecard reference stem cell lines.

(D) Heat map showing quantification of key embryonic stem cell and germ layer markers in iPSCs and following 8 days of undirected EB differentiation using the TaqMan hPSC Scorecard assay. Colours correspond to relative fold change (fc), or up- (red) and down- (blue) regulation, of genes.

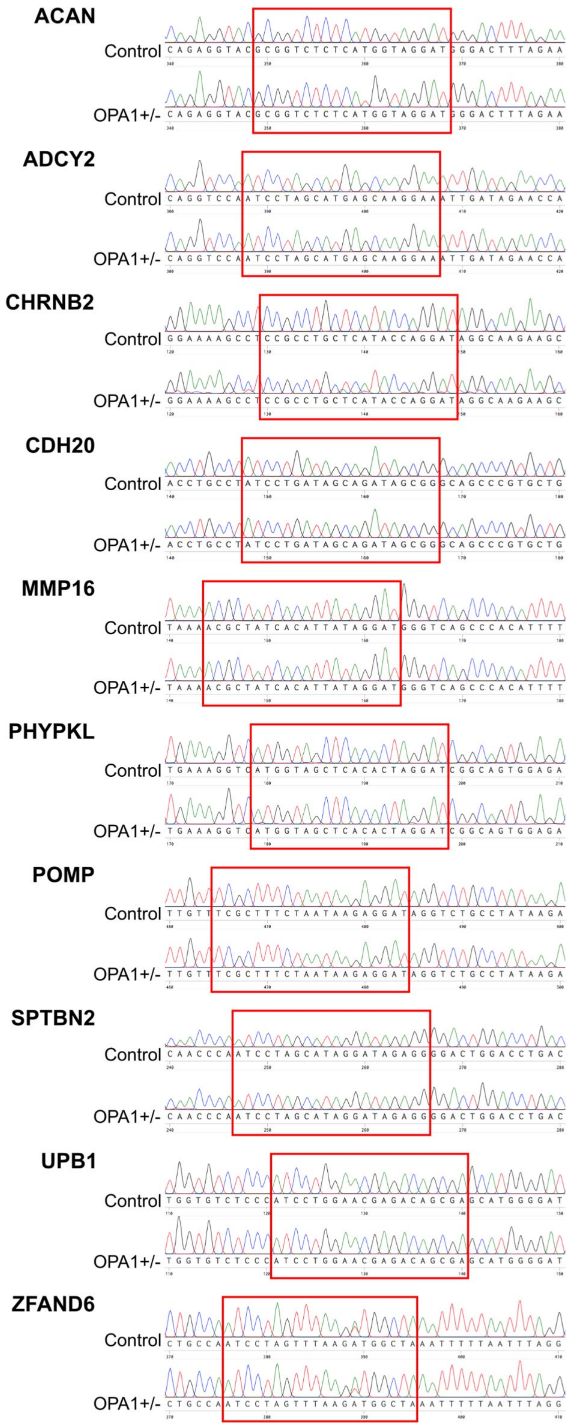

Figure S2. CRISPR/Cas9 off-target sequencing.

The top 10 predicted CRISPR/Cas9 off-target sites were determined using Off-Spotter. Targets were amplified by PCR for both WT and OPA1+/- edited iPSCs, followed by Sanger sequencing and alignment on Benchling.com. Red boxes denote predicted off-target gRNA sequence in control and OPA1+/- sequence traces. OPA1+/- displayed no sign of off-target CRISPR/Cas9 induced mutations.

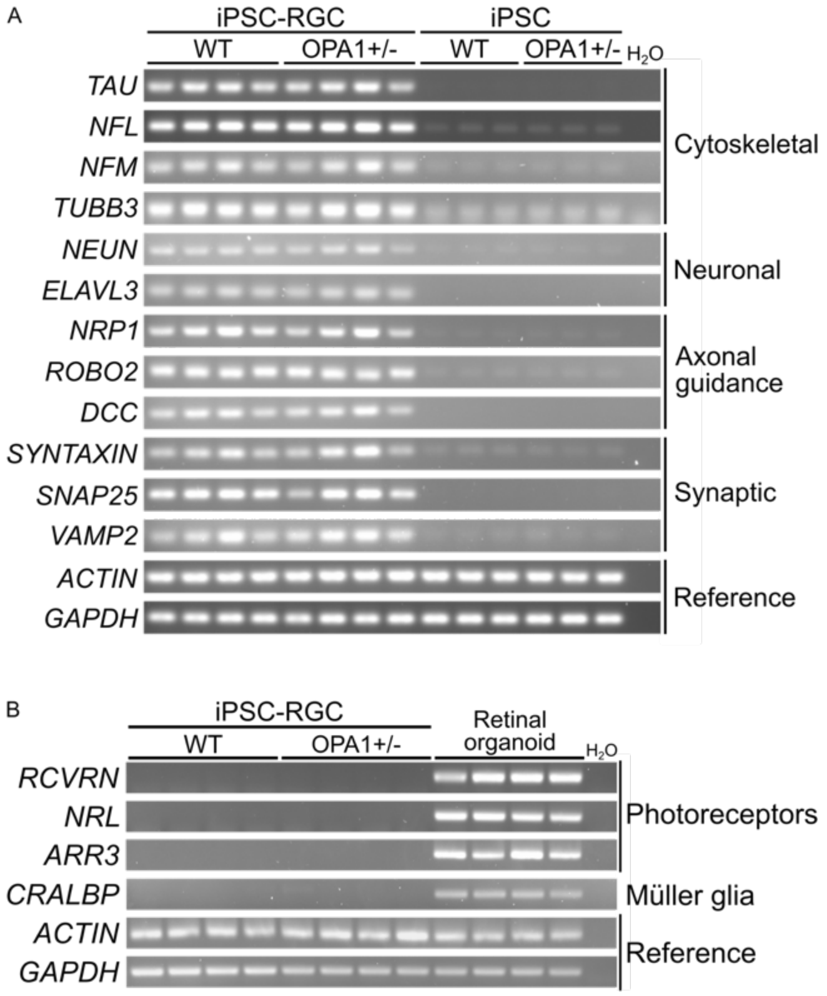

Figure S3. RT-PCR characterisation of D42 iPSC-RGC cultures.

(A) RT-PCR analysis of pan-neuronal genes associated with RGC maturation and function in D42 iPSC-RGCs and iPSCs. OPA1+/- iPSC-RGCs demonstrate no significant differentiation impairment for genes associated with the neuronal cytoskeleton (*TAU*, *NFL*, *NFM*, *TUBB3*), neuronal splicing (*NEUN, ELAVL3*), axonal guidance (*NRP1*, *ROBO2*, *DCC*) or synaptic function (*VAMP2*, *SNAP25*, *SYNTAXINI*). Each lane represents an independent iPSC-RGC differentiation or iPSC culture. *ACTIN* and *GAPDH* for reference. H_2O = water only control.

(B) iPSC-RGC cultures do not express markers of other retinal cell lineages. RT-PCR analysis of iPSC-RGCs using markers of photoreceptors (*RCVRN*, *NRL*, *ARR3*) and muller glia cells (*CRALBP*). cDNA from D90 retinal organoids used as positive control. *ACTIN* and *GAPDH* confirmed equal loading of cDNA. H_2O = water only control.

Table S1. CRISPR/Cas9 guide oligonucleotide sequences targeting *OPA1* exon 2.

Guide RNA (gRNA)	Guide oligonucleotide sequence	GC (%)	Number of predicted off-target genes (Off-Spotter)
gRNA 1	AATATGGCTACCAGCCTCGC	55	35
gRNA 2	TCGCTATCTCATACTAGGAT	40	85
gRNA 3	CATACTAGGATCGGCTGTTG	50	105

Table S2. Primers.

Gene	Method	Forward	Reverse	Size (bp)
ACAN	Off-target analysis	CAGAGAGGGGAGAGATTGATG	ATTTTGGAGGAGGGGGTTC	871
ACTIN	RT-PCR/ qPCR	CCAACCGCGAGAAGATGA	CCAGAGGCGTACAGGGATAG	97
ADCY2	Off-target analysis	GGACTGCTGGATTTCTCCGT	TTTCCTAGCCTTCCCAGCAG	600
ARR3	RT-PCR	CAGCTCAGCCCCTACAACTC	ACTGCAAAGCTCTGGGAGAA	244
ATOH7	qPCR	CTGCCTTCGACCGCTTACG	CAGAGCCATGATGTAGCTCAG	104
B2M	mtDNA qPCR	CACTGAAAAAGATGAGTATGCC	AACATTCCCTGACAATCCC	231
BRN3B (*POU4F2*)	qPCR	CAAGCAGCGACGCATCAAG	GGGTTTGAGCGCGATCATATT	157
CDH20	Off-target analysis	GACTATCCTCTTTCCTTCCTACACC	GCCTCACTCTACCACCTTTTG	443
CHRNB2	Off-target analysis	CTCACGCACACCCTACCAC	CTCTCCCCTAACCTCCAGCA	576
DCC	RT-PCR	ACCCAAGCTGGCTTTTGTACT	TGTGACGGCATCAGAAGGTTC	124
ELAVL3 (*HUC*)	RT-PCR	TCGAGTCCTGCAAGTTGGTTC	TGCATCATTGGGGTCAGAATAGT	86
GAPDH	mtDNA qPCR	CTCATCCAAGACTGGCTCCTCC	CAGCGTACTCCCCACATCAC	243
GAPDH	RT-PCR/ qPCR	CCCCACCACACTGAATCTCC	GGTACTTTATTGATGGTACATGACAAG	105
GNAT1	RT-PCR	CTGCTCACTCTGTCCCTTCG	TTGACGATGGTGCTCTTCCC	175
ISL1	qPCR	GCGGAGTGTAATCAGTATTTGGA	CACACAGCGGAAACACTCGAT	102
LHX2	qPCR	TCGGGACTTGGTTTATCACCT	GCAAGCGGCAGTAGACCAG	110
MMP16	Off-target analysis	AGATGTACCAAGAACCCCAAC	TGAGAAAAGAAAATGACTGCCAA	788
mtDNA 10kb	LR-PCR	CCCTCTCTCCTACTCCTG	CAGGTGGTCAAGTATTTATGG	9932
MTND1	mtDNA qPCR	ACGCCATAAAACTCTTCACCAAAG	GGGTTCATAGTAGAAGAGCGATGG	111
MTND4	mtDNA qPCR	ACCTTGGCTATCATCACCCGAT	AGTGCGATGAGTAGGGGAAGG	107
NEUN (*RBFOX3*)	RT-PCR	CCAAGCGGCTACACGTCTC	CGTCCCATTCAGCTTCTCCC	191
NFL	RT-PCR	TCAACGTGAAGATGGCTTTGGATA	AAGACCTGGGAGCTCTGGGAGTA	130
NFM	RT-PCR	ACAACCACGACCTCAGCAGCTA	ATGACGAGCCATTTCCCACTTT	86
NR2E3	RT-PCR	TGGTCCTCTTCAAGCCAGAGA	TTTCACCTCCACCCCCACTA	274
NRL	RT-PCR	CACTGACCACATCCTCTCGG	GAGGGTTCCCGCTTTACCTC	141
OPA1	qPCR	CGACCCCAATTAAGGACATCC	GCGAGGCTGGTAGCCATATTT	102
OPA1 ex2	Genotyping	GAGGTCTGCCAGTCTTTAGTG	TGTGTGAGAATGCCACCTGA	622
OPA1 ex27	Genotyping	TGGGTGATAAAACATACCAGGAG	GGAAGCTGGGTCAGGTATTG	1073
PAX6	qPCR	TCAGCTCGGTGGTGTCTTTG	GTCTCGGATTTCCCAAGCAA	328

PHYKPL	Off-target analysis	CACGAAGGGAAAGCAGAGG	CCAAGGAACAGAGGAGGAAT	753
POMP	Off-target analysis	CCATTTTTGCTGTTGACTTCC	TCCCTTGACTGACCCATTTG	918
RAX	qPCR	GTCTGTCGGTCCTGAGCAAA	ATATTGCGTCATGCCAGGGT	148
RECOVERIN	RT-PCR	AGCTCCTTCCAGACGATGAA	CAAACTGGATCAGTCGCAGA	150
RLBP1 (*CRALBP*)	RT-PCR	GCTGCTGGAGAATGAGGAAAC TC	GGCTGGTGGATGAAGTGGAT	174
ROBO2	RT-PCR	CTCTGCTACACTCACCGTCC	GCTGCCTTCTTTCTGCCAAA	145
SNCG	qPCR	TGAGCAGCGTCAACACTGTG	TGGCCTGTAGCCCTCTAGTC	187
SNAP25	RT-PCR	TCGTGTAGTGGACGAACGG	TCTCATTGCCCATATCCAGGG	158
SPTBN2	Off-target analysis	GAAACCCCGTCTCCACTAAA	GGTGCCCAGCCAAAATAAT	775
STX1A (*SYNTAXIN*)	RT-PCR	TAAAGAGCATCGAGCAGTCCA	GACATGACCTCCACAAACTTTCT	118
TAU	RT-PCR	CCAAGTGTGGCTCATTAGGCA	CCAATCTTCGACTGGACTCTGT	106
TUBB3	RT-PCR	TCAGCCGATGCGAAGGG	GGCCTCGTTGTAGTAGACGC	281
UPB1	Off-target analysis	AAATGCAAGTGGTTGCTCTGAC	TACTGCCACCTGACACACAAA	756
VAMP2	RT-PCR	TTGAAACAAGCGCAGCCAAG	ATGATGAGGATGATGGCGCA	97
VSX2	qPCR	GTGGCTACTGGGGATGCAC	TCCTGCTCCATCTTGTCGAG	108
ZFAND6	Off-target analysis	ACTGTGTCCATCGTGCCTG	TTCCTCATTTCTGCCCTTCCTTA	946

Table S3. CRISPR/Cas9 off-target regions.

Off-target genes were determined on Off-Spotter and amplified by PCR before Sanger sequencing. No off-target CRISPR/Cas9 induced mutations were identified. Red bases denote mismatches to *OPA1* gRNA 2.

Chromosome	Strand	Gene	Position	gRNA mismatches	Sequence	CRISPR/Cas9 induced mutation
15	+	ACAN	Intronic	5	gCGgTcTCTCATggTAG GAT	No
5	-	ADCY2	Intronic	5	TtcCTtgCTCATgCTAGG AT	No
1	+	CHRNB2	5' UTR	5	cCGCctgCTCATACcAG GAT	No
18	-	CDH20	Exon 11	5	cCGCTATCTgcTAtcAG GAT	No
8	-	MMP16	Intronic	4	aCGCTATCaCATtaTAG GAT	No
5	-	PHYKPL	Intronic	5	atGgTAgCTCAcACTAG GAT	No
13	+	POMP	Intronic	4	TCGCTtTCTaATAagAG GAT	No
11	+	SPTBN2	Intronic	5	cCtCTATCctATgCTAG GAT	No
2	-	UPB1	Exon 5	4	TCGCTgTCTCgTtCcAG GAT	No
15	-	ZFAND6	Intronic	4	TaGCcATCTtAaACTAG GAT	No

Table S4. Primary antibodies used for immunocytochemistry.

Antigen	Host	Clone	Isotype	Supplier/ Catalogue #	Working Concentration
BRN3A	Goat	C-20	IgG	Santa Cruz Sc-31984	500 ng/ml
BRN3A	Mouse	14A6	IgG_{2B}	Santa Cruz Sc-8429	4 μg/ml
BRN3B	Rabbit	Polyclonal	IgG	Abcam Ab56026	5 μg/ml
βIII TUBULIN (Neuronal)	Mouse	2G10	IgG_{2A}	Abcam Ab78078	1 μg/ml
ISL1	Rabbit	EP4182	IgG	Abcam Ab109517	4 μg/ml
NANOG	Mouse	23D23C6	IgG	Invitrogen MA1-017	6.6 μg/ml
Neurofilament 160/200 (NFH)	Mouse	RMdO20	IgG1	Sigma N2912	10 μg/ml
OCT4	Rabbit	Polyclonal	IgG	Abcam Ab19857	1 μg/ml
SNCG	Rabbit	Poly-clonal	IgG	Abcam Ab55424	5 μg/ml
SSEA4	Mouse	MC813	IgG3	Cell Signalling Technologies 4755S	3.3 μg/ml
TRA-1-61	Mouse	cl.A	IgM	Invitrogen 411000	1 μg/ml
TRA-1-80	Mouse	cl.26	IgM	Invitrogen 411100	1 μg/ml
